# Universal platform for quantitative analysis of DNA transposition

**DOI:** 10.1186/1759-8753-1-24

**Published:** 2010-11-26

**Authors:** Maria I Pajunen, Tiina S Rasila, Lotta J Happonen, Arja Lamberg, Saija Haapa-Paananen, Saija Kiljunen, Harri Savilahti

**Affiliations:** 1 Division of Genetics and Physiology, Department of Biology, Vesilinnantie 5, FIN-20014 University of Turku, Finland; 2 Institute of Biotechnology, PO Box 56, Viikinkaari 9, FIN-00014 University of Helsinki, Finland

## Abstract

**Background:**

Completed genome projects have revealed an astonishing diversity of transposable genetic elements, implying the existence of novel element families yet to be discovered from diverse life forms. Concurrently, several better understood transposon systems have been exploited as efficient tools in molecular biology and genomics applications. Characterization of new mobile elements and improvement of the existing transposition technology platforms warrant easy-to-use assays for the quantitative analysis of DNA transposition.

**Results:**

Here we developed a universal *in vivo *platform for the analysis of transposition frequency with class II mobile elements, i.e., DNA transposons. For each particular transposon system, cloning of the transposon ends and the cognate transposase gene, in three consecutive steps, generates a multifunctional plasmid, which drives inducible expression of the transposase gene and includes a mobilisable *lacZ*-containing reporter transposon. The assay scores transposition events as blue microcolonies, papillae, growing within otherwise whitish *Escherichia coli *colonies on indicator plates. We developed the assay using phage Mu transposition as a test model and validated the platform using various MuA transposase mutants. For further validation and to illustrate universality, we introduced IS*903 *transposition system components into the assay. The developed assay is adjustable to a desired level of initial transposition via the control of a plasmid-borne *E. coli *arabinose promoter. In practice, the transposition frequency is modulated by varying the concentration of arabinose or glucose in the growth medium. We show that variable levels of transpositional activity can be analysed, thus enabling straightforward screens for hyper- or hypoactive transposase mutants, regardless of the original wild-type activity level.

**Conclusions:**

The established universal papillation assay platform should be widely applicable to a variety of mobile elements. It can be used for mechanistic studies to dissect transposition and provides a means to screen or scrutinise transposase mutants and genes encoding host factors. In succession, improved versions of transposition systems should yield better tools for molecular biology and offer versatile genome modification vehicles for many types of studies, including gene therapy and stem cell research.

## Background

Transposable DNA elements constitute a class of discrete genome segments capable of moving from one genomic location to another [1]. These common genome residents are present in all kingdoms of life, being particularly abundant in eukaryotes [1,2], where they cover a sizeable fraction of the genome space (e.g., ~45% in human [3] and nearly 85% in maize [4]). Completed genome sequences demonstrate a wide diversity among mobile DNA and imply the existence of novel transposable element families yet to be discovered from a multitude of diverse life forms [2]. Mobile DNA elements produce genetic variation, supply material for genome innovations (e.g., new genes) and provoke genome instability [5], evidently earning them a highly significant role in the course of evolution.

Transposable elements can be exploited in many types of advanced genetic studies. Typical applications include insertional mutagenesis [6], genome manipulation [7], transgenesis [8], functional genomics studies [9,10], gene therapy [11,12] and generation of induced pluripotent stem cells [12]. Such methodologies are currently under strong development, and it is expected that novel transposition-based applications and new strategies will emerge in the near future.

Transposable elements which move via a DNA intermediate (i.e., class II elements or DNA transposons [1]) are widespread, both in prokaryotes and in eukaryotes. For transposition, they share a common overall reaction mechanism, albeit with some variation in details among different element families [13]. Characteristically, DNA transposons are mobilised by a machinery typically encoded by the elements themselves, and the most critical component is the catalytic protein, a transposase. Initiating transposition, the transposase binds sequence-specifically the transposon ends and, by synapsing the ends as a multimer, assembles a protein-DNA complex called a transpososome. Within the transpososome, transposase catalyses two chemical reactions, donor DNA cleavage and DNA strand transfer, ultimately attaching the transposon DNA to the target DNA. Because of the unity in their reaction mechanisms [13], similar research approaches and analytical methods can be used to study both prokaryotic and eukaryotic DNA transposons.

Bacteriophage Mu uses DNA transposition for propagation and encodes one of the most thoroughly characterised transposition machineries [14]. Despite the complexity of Mu transposition in natural contexts (e.g., certain auxiliary factors involved; see Discussion), a substantially simpler reaction can be performed *in vitro*. This minimal component reaction requires only a simple reaction buffer and three purified macromolecular components: MuA transposase, mini-Mu transposon DNA and target DNA [15]. The minimal system yields transposition products highly efficiently and with low target site selectivity [15,16]. In general, these properties make the Mu reaction ideal for a variety of advanced molecular biology [15,17-19], protein engineering [20-22] and genomics [9,23] applications. With an addition of an *in vivo *step, the minimal system can also be used for efficient gene delivery in bacteria, yeast and mammalian cells [24-26].

Various methods have been developed in the past for the *in vivo *analysis of transposition frequency, with typical examples including mating-out and phage assays [27,28], but these methods are not ideal for large-scale studies. Currently, the most widely used methods to study transposition *in vivo *exploit coloured microcolonies (papillae), growing within otherwise colourless bacterial colonies. Initially, such papillation assays were designed to score events involving transposon excision from a specific gene locus [29]. More recently, most papillation assays have exploited a reporter transposon, typically including *lacZ *as a marker gene. Insertion of the reporter transposon into a genomic locus under the control of an active promoter would generate a gene fusion, facilitating the expression of the *lacZ *gene, detectable as coloured Lac^+ ^papillae on colourless Lac^- ^colonies. The number of papillae per colony is proportional to the frequency of transposition events catalysed by the transposase. Useable papillation assays are currently available for a number of transposons, and typical examples include the assays for Tn*5 *[30], Tn*7 *[31], Tn*10 *[32], IS*903 *[33] and Mu [34]. All present-day papillation assays are characteristically element-specific, many of them lack adjustability and some are complicated by the use of several plasmids. Clearly, there exists a need for further methodology development, and a papillation assay that could eliminate the above limitations would be beneficial.

An increasing number of transposon systems have recently been adapted for advanced molecular biology and genomics applications, and their modification for better performance is under development. Concurrently, owing to genome projects, new transposons are being discovered essentially on a daily basis. Both improvement of the existing transposition applications and characterization of new transposition systems require robust quantitative assays. In this report, we describe a general *in vivo *platform for the quantitative analysis of transposition that should be applicable to a variety of mobile DNA elements.

## Results

**Figure 1:**
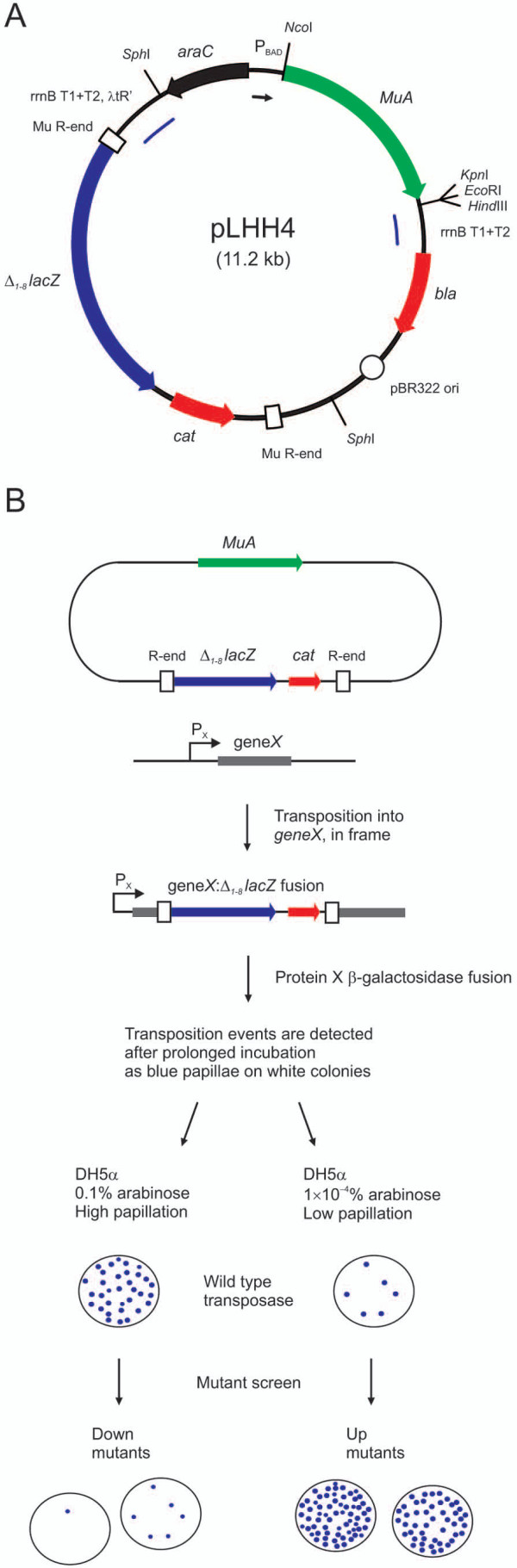
**Adjustable *in vivo *papillation assay**. (a) Papillation assay plasmid pLHH4. This plasmid contains the MuA transposase-encoding gene under the arabinose-inducible *E. coli *P_BAD _promoter, and the mobilisable mini-Mu reporter transposon Δ_1-8_LacZ-Cat-Mu. (b) Generation of papillae. The papillation assay plasmid is transferred into a phenotypically Lac^- ^*E. coli *strain. Expression of the transposase protein is induced by arabinose, mobilising the reporter transposon. Transposition of the element into an expressed gene (*geneX*) in the correct orientation and reading frame will form a *geneX::Δ_1-8_lacZ *gene fusion, and a β-galactosidase fusion protein will be expressed. Such transposition events can be detected on indicator plates after prolonged incubation as blue microcolonies on otherwise whitish colonies. The level of transposition can be adjusted by varying the transposase expression via changes in arabinose or glucose concentration.

A universal *in vivo *assay that could be used for the quantitative analysis of mobile DNA activity in various transposition systems would be a valuable tool for future transposon studies and application development. With this goal, we set up a platform that is based on the transformation of *E. coli *with a single plasmid driving transposon mobilization (Figure 1). The plasmid is engineered to enable straightforward construction of a reporter transposon for any specific DNA transposon system, and it contains a controllable unit for the inducible expression of the cognate transposase protein. The platform provides a visual read-out with blue microcolonies (papillae) growing within otherwise whitish bacterial colonies and allows the adjustment of the transposition frequency to a quantifiable range. As a test system for the platform development, we utilized bacteriophage Mu transposition. For further assessment of the assay and for the demonstration of its universality, we additionally employed IS*903 *transposition.

### Papillation assay

Initially, we constructed the papillation assay plasmid pLHH4 (Figure 1a), which encodes the phage Mu transposase and, in addition, contains a mobilisable mini-Mu reporter transposon. This pBR322-derived plasmid exhibits medium copy number and encodes β-lactamase as a standard selectable marker. The plasmid also includes the arabinose/glucose-controllable P_BAD _promoter of *E. coli*, driving the inducible expression of MuA transposase. The expression unit is bordered by strong transcription terminators to prevent surplus gene expression from sequences flanking the unit. The mini-Mu transposon (Δ_1-8_LacZ-Cat-Mu) contains, as an inverted repeat, a 50-bp segment of Mu R-end DNA in each of the transposon ends. As a reporter gene, the transposon contains a promoterless, 5'-terminally truncated *E. coli lacZ *gene (*Δ_1-8_lacZ*), lacking the codons for amino acids 1-8. The transposon also includes the *cat *gene for chloramphenicol selection.

The papillation assay for Mu transposition takes advantage of the introduction of the plasmid pLHH4 into a phenotypically Lac^- ^*E. coli *strain, and the plasmid facilitates the reporter transposon mobilisation upon sufficient transposase expression. Successful transposition into an expressed chromosomal gene in the appropriate orientation and reading frame gives rise to *lacZ *gene fusions. These events can be detected as blue papillae on a colony grown on an indicator plate containing X-gal, with each papilla representing an independent transposition event (Figure 1b).

### Colony phenotypes and papillation characteristics of different *E. coli *strains

We evaluated three *E. coli *strains: DH10B, DH5α and JM109 (Additional file 1) with regard to their suitability for the papillation assay. These standard laboratory strains are phenotypically Lac^-^, and they grow as whitish colonies on X-gal indicator plates. As a low-papillation-frequency control, we also included strain HT321 in the evaluation. This strain harbours a mutation in the *pcnB *locus, resulting in a reduced plasmid copy number [35], and has previously been used to identify host mutations that increase transposition frequency in IS*903*, Tn*10 *and Tn*552 *systems [33]. Plasmid pLHH4 was transformed into the cells, and colonies were grown for a 115-h reference time period on a standard papillation medium with varying arabinose concentrations (Table 1). The critical characteristics evaluated were: (1) colony size, because large colonies would potentiate a broad dynamic range for the assay; (2) the number of papillae generated should be adjustable by arabinose over a wide range of concentrations; (3) a reasonable maximum transposition frequency should be attained; (4) clearly defined papillae should be evenly distributed on individual colonies; and (5) the number of the papillae per colony should not be overly influenced by the number of growing bacterial colonies on a given plate.

**Table 1:** Colony phenotypes and papillation characteristics in different *E. coli *strains

Strain	Colony size^a^	Adjustabilitywith arabinose	Maximum frequencyof transposition^b^	Distribution ofpapillae on colonies	Papillae number dependency on the number of growing colonies per plate^c^
DH10B	Large	Limited	High (up to ~100 papillae)	Centered in the middle	Somewhat dependent
DH5α	Large	Good	Highest (up to ~300 papillae)	Even	Largely independent
JM109	Medium	Good	Intermediate (up to ~40 papillae)	Even	ND^d^
HT321	Large	Moderate	Low (up to ~10 papillae)	Even	ND

^a^Large colony size: up to ~5 mm of diameter. Medium colony size: up to ~3 mm of diameter.

^b^The maximum frequency of transposition is specified in this table as the number of papillae per colony grown at 30°C for 115 h on a standard papillation medium containing 0.1% arabinose.

^c^See Additional file 2.

^d^ND, not determined.

Strain DH10B formed large colonies with a convex center and generated a relatively high maximum number of papillae. The number of papillae generated responded to the increase in the arabinose concentration, but not linearly, and two maxima were observed (at 0.001 and 1% arabinose concentrations; data not shown). The reason for this somewhat unexpected behaviour is not known but might possibly point to unknown alterations in the arabinose transporter systems (see Discussion). Most papillae were distributed in the center of the colony, and the number of papillae on even-sized colonies was somewhat influenced by the number of growing colonies per plate (Additional file 2).

Strain DH5α formed large flat colonies, and the number of papillae correlated well with the arabinose concentration in the growth medium. A high maximum number of clearly defined papillae formed throughout a colony. The number of growing colonies per plate did not overly influence the frequency of transposition. Particularly within the range of 40 to 200 colonies per plate, papillae formed with a very similar rate (Additional file 2).

Strain JM109 generated medium-size, irregularly shaped colonies. The number of papillae on a colony was proportional to the arabinose concentration used, reaching the level of ~40 papillae per colony upon 115-h incubation. Up to ~120 papillae per colony formed with a prolonged (164 h) incubation time (data not shown). Clearly defined papillae formed throughout a colony, but curiously, ~7% of the colonies appeared completely white, thus exhibiting Lac^- ^phenotype; the same clones were also proline auxotrophs (data not shown). These data point to the loss of the resident F'-episome, which in this strain encodes the critical lactose utilization and proline biosynthesis proteins.

The control strain HT321 generated large colonies and with the 115-h reference incubation time exhibited a low transposition frequency (up to ~10 papillae per colony), too low to unambiguously evaluate arabinose responsiveness. However, with prolonged incubation time (164 h), up to ~30 papillae per colony could be observed, and arabinose dependency was apparent (data not shown).

### The effect of temperature on transposition frequency

**Figure 2:**
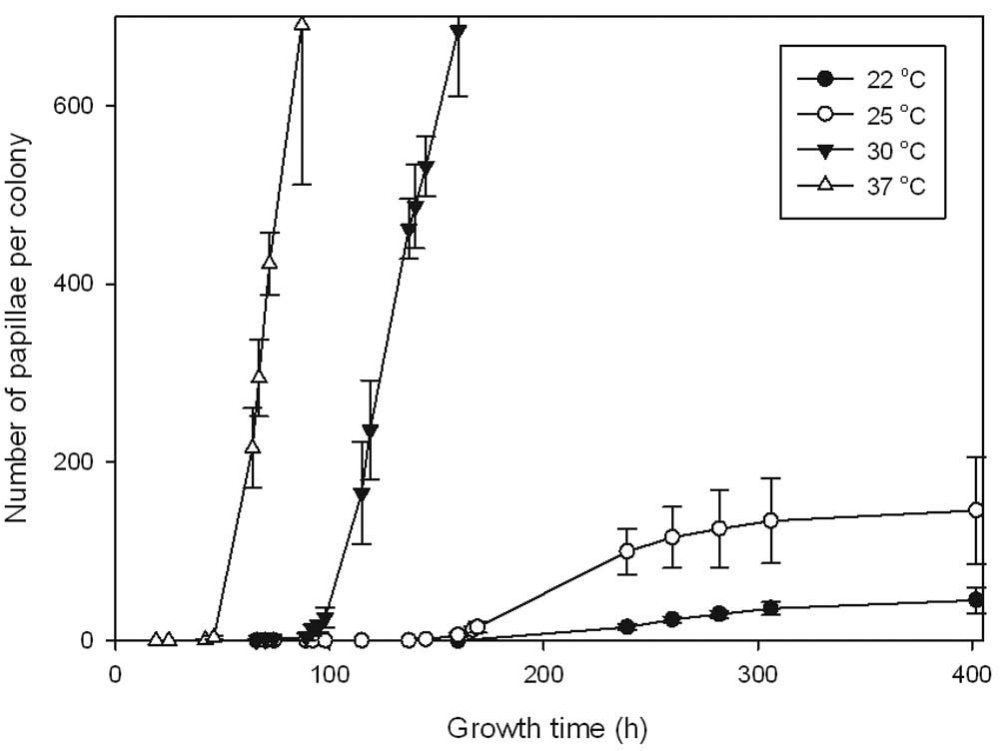
**The effect of temperature on transposition frequency depending on temperature**. The arabinose content in the growth medium was 0.1%. The transposition frequency at 37°C is shown with an open triangle, at 30°C with a filled triangle, at 25°C with an open circle and at room temperature (22°C) with a filled circle, respectively. Papillae were enumerated from six colonies for each data point. The error bars indicate SD above and below the average value for each data point.

Strain DH5α appeared ideal for the further characterization of the papillation assay. We next examined the effect of growth temperature on papillation. DH5α cells carrying the plasmid pLHH4 were grown on papillation plates in standard growth conditions (0.1% arabinose) at four different temperatures: 22°C (room temperature), 25°C, 30°C and 37°C. Emerging papillae were enumerated as a function of incubation time (Figure 2). At each temperature, visible papillae began to appear on colonies after a prolonged incubation period, and the growth rate of the bacteria apparently dictated the time course of the appearance. The two lower temperatures produced a modest number of papillae with a plateau in their appearance. In contrast, the two higher temperatures generated papillae in increasing numbers. Yielding a measure for the maximum dynamic range of the assay, we were able to enumerate papillae up to a time point where ~700 discernible blue spots per colony were visible; thereafter the papillae fused, and the entire colony turned blue. At 30°C and 37°C, papillae formation appeared linear between the time points ~100-150 h and ~50-80 h, respectively. For further studies, we chose to use incubation at 30°C, as at this temperature papillae accumulated more slowly, allowing a more convenient and accurate papillae enumeration.

### MuA expression and transposition frequency

The expression level of MuA transposase should influence the transposition frequency measurable by the papillation assay. We therefore studied the MuA expression level and transposition frequency under variable inducer concentrations. The former was studied using liquid cultures and the latter, in a separate experiment, using papillation analysis on plate cultures.

**Figure 3:**
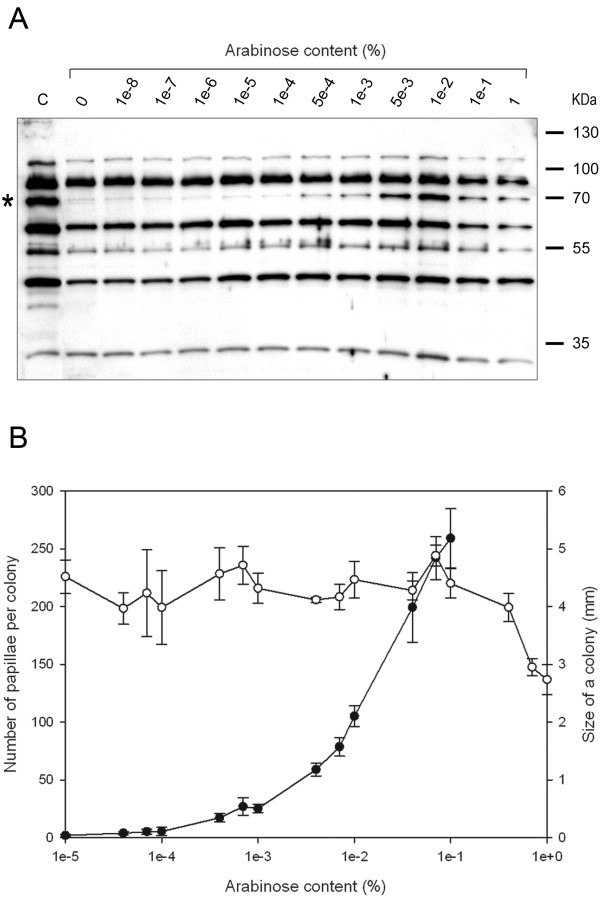
**The effect of growth conditions on transposition frequency**. (a) Western blot analysis of MuA expression levels at varying arabinose concentrations. Whole cell lysates from *E. coli *DH5α cells containing plasmid pLHH4 were analysed for MuA content using polyclonal rabbit anti-MuA antibody. The asterisk indicates MuA. (b) The effect of arabinose concentration on colony size and papillation. Wild-type MuA-expressing plasmid (pLHH4) was used for papillation analysis with *E. coli *DH5α as a plasmid recipient strain. Open and filled circles indicate the colony size and the number of papillae per colony, respectively. Papillae were enumerated from six colonies for each data point. The error bars indicate SD above and below the average value for each data point.

The initial MuA expression analysis was done with whole-cell lysates of liquid cultures grown under variable arabinose concentrations. SDS-PAGE and Western blotting were used to reveal population-average protein expression levels of MuA (Figure 3a). The data indicated that MuA expression was increased by the increase in the arabinose concentration up to 1 × 10^-2^%, and the induction was detectable already at 5 × 10^-4^%. Altogether, the results demonstrated that the inducible expression system was functional and adjustable over a large range of arabinose concentrations. The apparent reduction in MuA expression at greater than 1 × 10^-2^% arabinose concentrations in this experiment is difficult to explain, but it may conceivably relate to MuA overexpression possibly causing harmful effects on various cellular functions and ultimately resulting in differences in the growth rate.

Transposition frequency was subsequently analysed under variable arabinose concentrations by the papillation assay (Figure 3b). Papillae began to appear at ~1 × 10^-4^% arabinose concentration, and the highest number of discernible papillae was obtained at 1 × 10^-1^% concentration. Higher arabinose concentrations apparently affected cell survival significantly, as colonies were smaller (Figure 3b) and ragged (data not shown). Furthermore, at these concentrations, the papillae were fuzzy with diffuse colour and impossible to enumerate. These data indicated that measurable papillation could be induced over a wide range of arabinose concentrations.

In general, the MuA expression levels observed from liquid cultures correlated well with the papillation data obtained from plate cultures. However, some discrepancy was seen at 1 × 10^-1^% arabinose concentration (Figure 3a and 3b). This latter result likely reflects key differences between the analyses, liquid versus plate cultures. In liquid cultures, the ambient arabinose concentration should be equal for all cells, and this is not necessarily the case for cells growing as colonies on agar plates. With regard to the arabinose concentration, the microenvironment within a colony on a plate may vary for each individual cell. Furthermore, the actual arabinose concentration experienced by the cells within a colony conceivably is, in general, somewhat lower than that in the medium because of diffusion characteristics and colony morphology.

### The effect of MuA activity on transposition frequency

**Figure 4:**
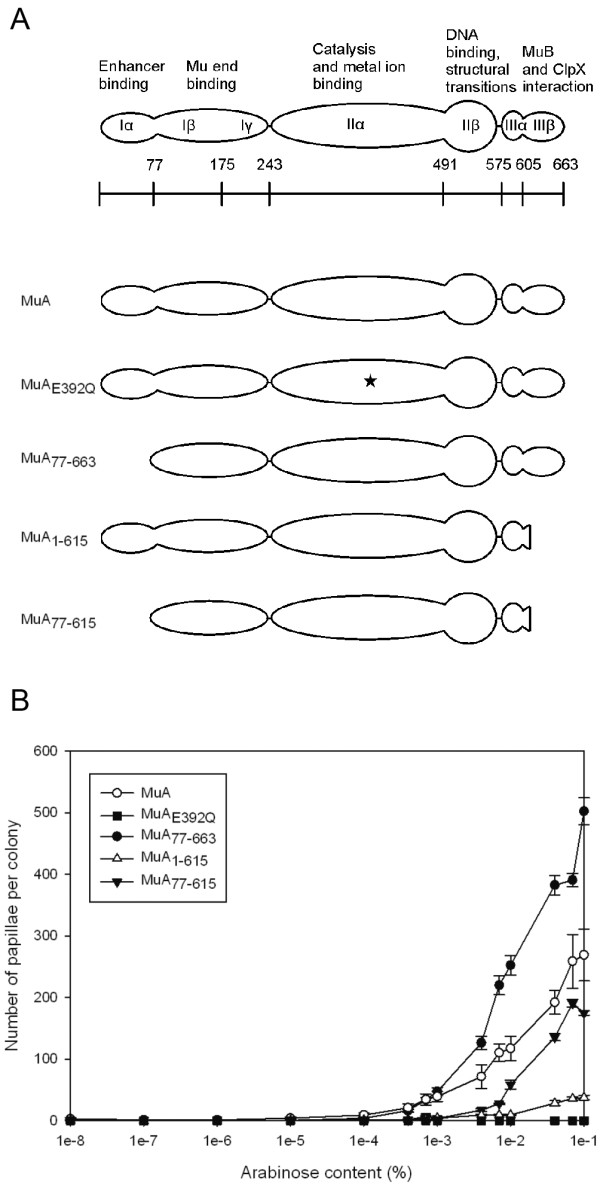
**Papillation analysis of different MuA variants at varying arabinose concentrations**. (a) MuA proteins used in papillation analysis. Structural organisation of MuA with different functions assigned to various domains [14]. Amino acid numbers corresponding to the amino terminus of each subdomain are shown beneath the structure. (b) *In vivo *transposition frequency induced by MuA variants. The transposition frequency of MuA_77-663 _is shown with a filled circle, wild-type MuA with an open circle, MuA_77-615 _with a filled triangle, MuA_1-615 _with an open triangle and MuA_E392Q _with a filled square, respectively. Papillae were enumerated from a total of three to six colonies for each data point. The error bars indicate SD/2 above and below the average value for each data point.

The suitability of the papillation assay for the quantitative comparison of variable transposition frequencies was examined using several MuA variants (Figure 4a). We used an N-terminally deleted MuA_77-663 _as well as C-terminally deleted variant MuA_1-615_, and in addition, a variant missing both termini MuA_77-615_. We also used a MuA variant with a substitution in the catalytically important active site DDE-triad (MuA_E392Q_), a protein that is proficient for the transpososome assembly but catalytically defective [36]. Standard papillation conditions with 115-h incubation time were used in these experiments.

**Figure 5:**
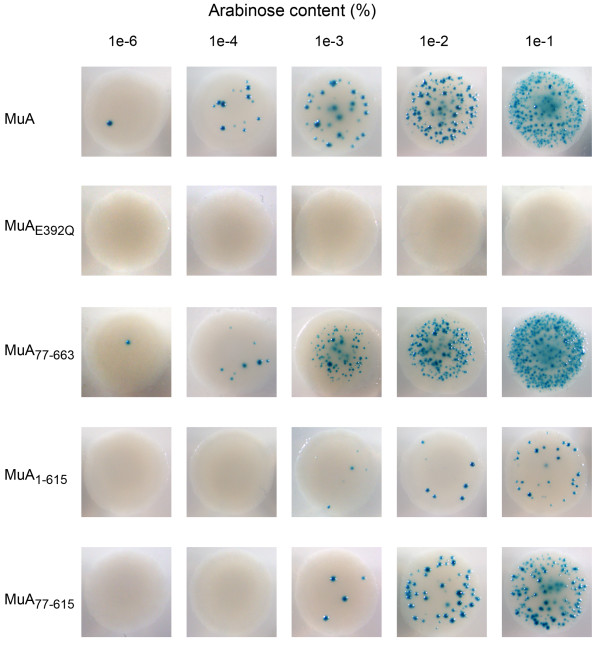
**Colonies from papillation assay**. Papillation of MuA variants in DH5α at varying arabinose concentrations.

The wild-type MuA and all of the deletion variants studied produced papillae and responded to the increase in the arabinose concentration (Figure 4b and Figure 5). The wild-type protein produced ~300 papillae per colony at the highest arabinose concentration tested (0.1%). Both MuA_1-615 _and MuA_77-615 _produced significantly less papillae with maximum yields of ~50 and ~200 papillae, respectively, indicating hypoactivity. MuA_77-663 _maximally gave rise to ~500 papillae and thus appeared hyperactive. The active site mutant MuA_E392Q _did not produce papillae in the assay, most probably directly reflecting its reported deficiency in catalysis. Similarly, no papillae were generated when a pLHH4 derivative (pTLH1), missing the *MuA *gene but retaining the Mu ends, was assayed as a control (data not shown), highlighting the specificity of the system. These results indicate that variable transposition activity levels can be quantified reliably, demonstrating the suitability of the papillation assay to screen transposase mutants with altered activities. The data also show that the transposition frequency can be fine-tuned to a desired initial level for screening purposes by the adjustment of the arabinose concentration in the medium.

We include a time-lapsed video of growing colonies from a papillation analysis (Additional file 3), which highlights graphically the characteristics the developed assay. The video was recorded using papillation conditions where wild-type MuA produced only a few papillae (1 × 10^-4^% arabinose). Using these assay conditions, we have recently identified a number of hyperactive MuA variants, and a comprehensive analysis of critical amino acid changes involved in their hyperactivity phenotype will be described in a future report.

### Universal plasmid for papillation analysis

We next modified the papillation assay plasmid pLHH4 to be useful with other DNA transposons. In this modified plasmid, pSKT1 (Additional file 4), the Mu R-ends have been replaced by polylinkers, allowing a straightforward directional cloning of heterologous transposon ends. Similarly, the MuA transposase gene is also replaced by a polylinker, facilitating directional cloning of a heterologous transposase gene. The functionality of the universal system was then confirmed as follows: (1) Mu R-ends were recloned into the appropriate newly made linker sites and tested in the context of wild-type *MuA*; a similar number of papillae were generated, as was observed with the original papillation assay plasmid pLHH4 (data not shown). (2) When the Mu R-ends were omitted, no papillae were produced, further illustrating the specificity of the assay system (similar to the omission of the MuA transposase gene discussed above). (3) Finally, the components of the IS*903 *transposition system were introduced into pSKT1, and the ensuing plasmid pSKT4 (Additional file 5) was analysed for transpositional activity (results described in the next subsection).

### Analysis of IS*903 *transposition

**Figure 6:**
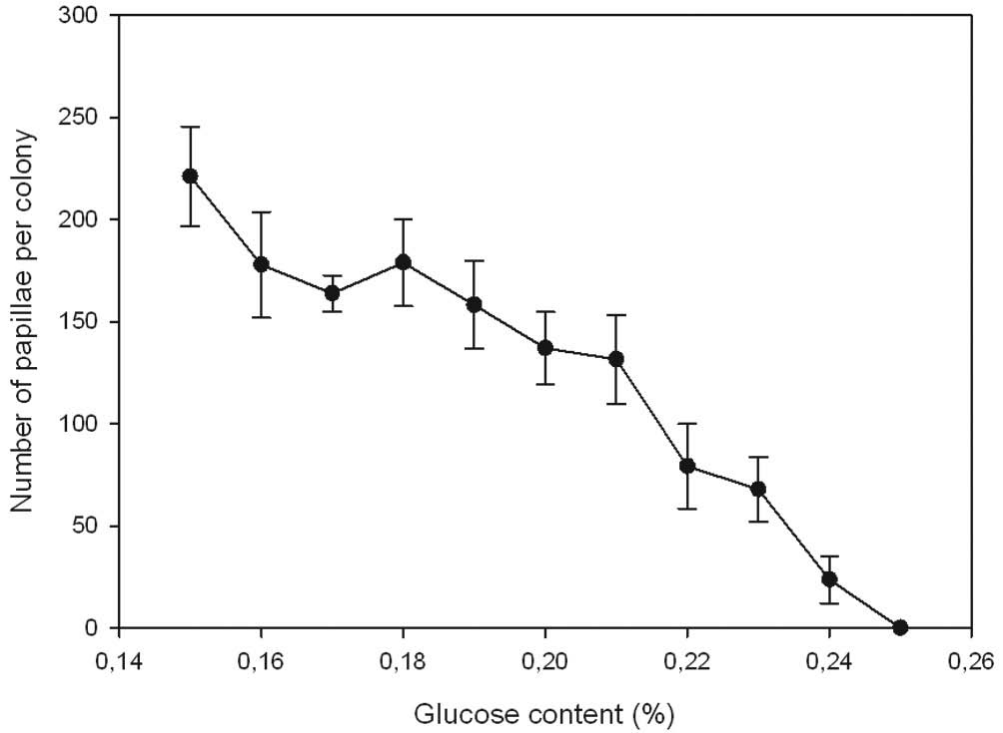
**Papillation analysis of IS*903 *transposition**. Plasmid pSKT4 was introduced DH5α cells for papillation at varying glucose concentrations. Papillae were enumerated from six colonies for each data point. The error bars indicate SD above and below the average value for each data point.

Plasmid pSKT4, containing the critical components of the IS*903 *transposition system, was introduced into DH5α cells and analysed for papillation with variable concentrations of arabinose in the growth medium. All tested concentrations yielded papillae, and somewhat surprisingly, even the plates which did not contain arabinose produced ~200 papillae per colony (data not shown), indicating that IS*903 *transposase was expressed at levels sufficient for transposition. We next tested whether glucose, conceivably via catabolite repression, could be used to tighten the control of transposase expression, which in turn would result in reduced levels of papillation. A constant number of ~200 papillae were produced with glucose concentrations up to 0.15% (data not shown). However, higher concentrations of glucose reduced the number of papillae essentially linearly, and upon 0.25% concentration, no papillae were produced (Figure 6). Thus, in cases where uninduced transposase expression results in transposition frequency that is too high for screening purposes, glucose can be used to fine-tune papillae formation to a desirable level.

## Discussion

Papillation assay systems have provided straightforward means to dissect transposition events *in vivo*. For example, they have enabled simultaneous activity analyses of tens of thousands of randomly generated transposase mutants [31,37-40]. Furthermore, such assays have successfully been used to study the effects of mutations in transposon end sequences [30,34,41-43] and to examine the influence of host factors [33]. Although each particular assay system has performed adequately in the context in which it has been used, in many cases the adjustability has been limited. Furthermore, many of the assays have included a reporter transposon and the cognate transposase gene separately in different plasmids, which is not an ideal arrangement for many applications.

The system we describe provides certain advantages. First, the assay is based on an easy-to-use single plasmid, and it offers a straightforward means to visually screen mutant versions of transposases as well as potentiates the discovery of host factors affecting transposition. Second, the inclusion of two antibiotic marker genes in the assay plasmid, *bla *and *cat*, enables double antibiotic selection, which effectively prevents the growth of satellite colonies on plates during the long incubation period needed for papillation. Note that the *cat *marker gene within the reporter transposon can also be used for the selection of transposition events, a useful feature in prospective downstream applications such as mating-out assays. Third, the transposition frequency is easily adjustable, which potentiates many types of studies under a desired optimal rate of transposition.

The expression system used in our assay, the arabinose-controllable induction via P_BAD _promoter, has been studied in detail previously [44]. The study revealed that with standard *E. coli *strains harbouring native arabinose transporter systems *araE *and ara*FGH*, the arabinose-inducible P_BAD _promoter is subject to essentially all-or-none induction via a regulatory feedback loop, in which limiting arabinose concentrations give rise to subpopulations of cells that are either highly induced or uninduced [44]. Raising the arabinose concentration increases the fraction of highly induced cells until a saturating concentration is reached, under which concentration essentially all of the cells in the population become highly induced [44]. Nevertheless, even under these saturating arabinose concentrations, a degree of variation exists among the expression levels of individual cells [44]. Considering the above features, it was expected that in our assay system, only a fraction of cells would express the transposase within any given colony under subsaturating arabinose concentrations, and those cells expressing the transposase would be the ones which ultimately produced the observed papillae. Further considering the above features, although all-or-none induction should generate two subpopulations of cells, a degree of transposase expression variation should still be present within these subpopulations in both subsaturating and saturating arabinose concentrations. Overall, our results have shown that good control of population level average transposase expression can be achieved using the P_BAD _promoter, at least under subsaturating arabinose concentrations. Under these conditions, the transposase expression levels correlate very well with the formation of papillae, forming a basis for a dependable quantitative assay.

The omission of arabinose on selection plates effectively eliminated transposition in the Mu system, but identical conditions resulted in a reasonable level of transposition in the IS*903 *system. These data indicate that the P_BAD _control unit of transposase expression in the papillation assay plasmid allows some basal protein expression. As it is likely that similar expression levels would be achieved independently of the expressed protein, the observed differences between Mu and IS*903 *most probably reflect differences in the inherent activities of the two systems (in the conditions tested) rather than differences in the transposase expression levels. The observed activity differences are necessarily not surprising, because the analysed Mu system is somewhat aberrant (unnatural transposon end configuration, lack of transposition enhancer, lack of MuB protein), whereas the analysed IS*903 *transposition represents a wild-type system.

We examined several *E. coli *strains for their suitability in the papillation assay. From these studies, it became evident that a number of critical attributes affecting the assay performance were strain-dependent, evidently reflecting known (plus possibly unknown) genotypic differences, and by all criteria DH5α appeared optimal for the assay. First, the large colony size and the even distribution pattern of papillae within a colony allowed scoring of more than 500 discernible papillae. Second, the adjustability of the assay spans a wide range of arabinose concentrations. Together, these attributes generated a large dynamic range for the assay, and the use of glucose extended the adjustability even further. Third, the colony density on a plate did not influence papillation appreciably, in practice meaning that reliable results could be obtained from standard plates (diameter 9 cm) containing ~50-200 growing colonies.

Several MuA variants were scrutinised for their transpositional activity to test the assay under different levels of transposase activity. Overall, the results showed that activities above and below a certain (in this case, wild-type MuA) activity level could reliably be analysed, potentiating the screen for hyperactive and hypoactive mutants (Figure 4b and 5). MuA_77-663_, missing the Iα domain [45], has been shown to be hyperactive in certain *in vitro *assays [46]. Similar hyperactivity was also seen in its proficiency to produce papillae. MuA_1-615_, lacking the C-terminal 48 amino acids [47], retains a wild-type level of activity in certain *in vitro *assays [47,48]. However, in our *in vivo *analysis, it was clearly hypoactive. The details behind the apparent discrepancy are unknown but may reflect the deficiency in the disassembly of the transpososome via ClpX-mediated interaction, a property confined to the C-terminus of MuA [14]. MuA_77-615_, having both the Iα domain and the C-terminal residues deleted, retained substantial activity. Thus, the hyperactivity-causing property of the domain Iα deletion appeared to compensate for the activity reduction caused by the deletion in the C-terminus.

Mu transposition can be reproduced *in vitro *using as sole components MuA transposase, transposons that contain 50 bp of Mu R-end in each transposon terminus, and target DNA. This minimal reaction forms the basis of the Mu *in vitro *transposition technology [15]. With an overall aim to improve the technology, we designed our assay system to mimic this minimal *in vitro *transposition reaction as closely as possible. Accordingly, we used a reporter transposon which contains two R-ends and did not include in the system the known auxiliary factors of Mu transposition, such as the transposition enhancer (IAS) and activator/targeting protein MuB [14]. Our results demonstrate that Mu transposition *in vivo *can proceed efficiently independent of the natural L/R (left/right) end configuration and in the absence of the above auxiliary factors. While a massive number of Mu studies have employed the natural L/R end combination for studies *in vivo*, significantly less *in vivo *data are available with regard the L/L or R/R end configurations. Nevertheless, these configurations have been shown to promote transposition *in vivo*, albeit with reduced efficiency, and to generate correct junctions between the Mu DNA and host DNA (unpublished data in [49]). Falling outside the scope of the current study, we did not analyse the effects on transposition of the known *E. coli *host factors, HU and IHF. Nevertheless, it would be interesting to see whether these two proteins were dispensable under the described *in vivo *conditions as well.

The papillation assay platform should be applicable to a number of transposition systems. For cut-and-paste type transposons, papillation assays generate gene fusions by the initial detachment of the transposon from the donor site, followed by the integration of the transposon into another locus. Thus, the mechanistic basis of the assay for cut-and-paste transposons is relatively simple. However, Mu and IS*903 *primarily use the replicative mode of transposition, a somewhat more complicated mechanism. During replicative transposition, a branched transposition intermediate (a/k/a Shapiro intermediate) is formed. This intermediate can be processed either by DNA replication or by DNA repair, and it is known that Mu uses both of these pathways for processing [50]. However, in this work, we have not studied the mechanistic details of the assay. Recent findings indicate that several pathways may be involved in the processing of the Shapiro intermediate [50], which necessarily would complicate such studies. As retroviruses and LTR retrotransposons catalyse a mechanistically related (transposition) reaction called integration [1], it might be possible to apply the assay platform also for them. However, in these cases, the interplay between the integrase protein and other proteins of the integration machinery may complicate the development of a useable strategy.

## Conclusions

We have established a papillation assay platform which is easily modifiable for a variety of transposon systems. It provides an efficient tool for the studies of yet unknown transposons and yields means to screen and scrutinise transposase mutants as well as genes encoding critical host factors involved in transposition. Although in this paper we used the papillation assay with two prokaryotic transposons, the system may equally be applied to eukaryotic DNA transposons as well. It is hoped that the use of the developed platform will generate better versions of transposition systems, ultimately producing better tools for molecular biology and offering versatile genome modification vehicles for a variety of future studies. These investigations are expected to involve disciplines such as cancer biology, gene therapy, stem cell research and studies on transgenesis.

## Methods

### Bacteria, plasmids and oligonucleotides

*Escherichia coli *strains are listed in Additional file 1. Plasmids and oligonucleotides are described in Additional file 6 and Additional file 7, respectively.

### Reagents, enzymes and DNA techniques

Ampicillin, chloramphenicol, arabinose and glucose were from Sigma (St. Louis, MO, USA). Lactose was from BDH/VWR International (West Chester, PA, USA) and 5-bromo-4-chloro-3-indolyl-β-D-galactopyranoside (X-gal) was from AppliChem GmbH (Darmstadt, Germany). Restriction endonucleases and T4 DNA ligase were from New England BioLabs (Ipswich, MA, USA). Calf intestinal phosphatase (CIP) was from Finnzymes (Espoo, Finland). Standard DNA techniques were performed as described previously [51]. Plasmids were propagated in *E. coli *DH5α and were isolated using appropriate Qiagen (Hilden, Germany) kits. Electrocompetent DH5α cells were prepared as described previously [24] and used for standard cloning procedures.

### Construction of the papillation assay plasmid expressing wild-type MuA transposase

The papillation assay plasmid pLHH4 (Figure 1a), expressing wild-type MuA transposase (GenBank accession number P07636) was generated as follows: (1) A truncated version of the *lacZ *gene, encoding *E. coli β*-galactosidase (EC 3.2.1.23) but missing the nucleotides specifying the amino acids 1-8 (*Δ_1-8_lacZ*), was amplified by polymerase chain reaction (PCR) from plasmid pNT105 using the primer pair HSP173/HSP408. The generated PCR fragment was trimmed with *Bam*HI and subsequently cloned into plasmid pSupF-Mu between the two *Bam*HI sites to replace the *supF *gene, yielding plasmid pLHH1. (2) The *cat *gene was amplified by PCR from plasmid pBC SK(+) using the primer pair HSP360/HSP361. The generated PCR fragment was trimmed with *Bam*HI and cloned downstream of *Δ_1-8_lacZ *into *Bam*HI-linearized pLHH1 (partial digestion), yielding plasmid pLHH2. Prior to cloning, the *cat *gene was modified by introducing, via overlap PCR with appropriate primers, a silent mutation (codon 177, Thr, ACC→ACT) to eliminate a critical *Nco*I site. (3) An *Sph*I-fragment containing the tandem *E. coli *rrnB terminators T1+T2 [52] and phage λ tR' terminator [53] as well as a critical *Bam*HI site for cloning was generated by standard DNA techniques including PCR and annealing of oligonucleotides (see Additional file 7). (4) The above terminator-containing *Sph*I-fragment was then cloned into *Sph*I-digested pALH6 to generate pLHH3. (5) Finally, the mini-Mu transposon of pLHH2 was released with *Bgl*II and cloned into *Bam*HI-linearised pLHH3 (partial digestion) to generate pLHH4.

### MuA variant-expressing papillation assay plasmids

Initially, plasmid pTLH1 was generated by cloning the 5.2-kb *Sph*I reporter-containing fragment of pLHH4 into *Sph*I-digested pBADHisA, which substituted the *MuA *gene with the polylinker of pBADHisA. PCR-generated *MuA *deletion variants (pMPH14, pMPH17, pMPH18; see Additional files 6 and 7) were subsequently cloned between the *Nco*I and *Xho*I sites of pTLH1. The plasmid expressing *MuA_E392Q _*(pLHH12) was generated by cloning the *Nco*I-*Bam*HI fragment of pMK616 into pTLH1 digested with *Nco*I and *Bgl*II.

### Construction of universal papillation assay plasmid

Initially, the reporter region of pLHH2 containing the *Δ_1-8_lacZ *and *cat *genes was amplified by PCR using the linker-sequence-tailed primers HSP685 and HSP689 (see Additional file 7). The generated PCR fragment was trimmed with *Bgl*II and cloned into *Bam*HI-linearized pLHH3 (partial digestion), yielding plasmid pMPH23B. Finally, the universal papillation assay plasmid pSKT1 (Additional file 4) was obtained by replacing the *MuA*-containing *Nco*I-*Sca*I fragment of pMPH23B with the polylinker-containing *Nco*I-*Sca*I fragment of pBADHisA. Plasmid pSKT1 can be applied to establish a papillation assay for various transposon systems in three straightforward cloning steps involving transposon ends and a cognate transposase gene.

### Construction of papillation assay plasmid for IS*903*

The IS*903 *transposase gene was amplified by PCR from plasmid pNT105 using the primer pair HSP693/HSP694. The PCR fragment was trimmed with *Nco*I and *Xho*I and cloned into plasmid pSKT1 digested with the same two enzymes, yielding plasmid pSKT2. The ends of IS*903 *were then inserted as follows. First, oligonucleotides HSP690 and HSP691 were annealed and cloned into pSKT2 digested with *Spe*I and *Not*I, resulting in plasmid pSKT3. Second, oligonucleotides HSP690 and HSP692 were annealed and cloned into pSKT3, digested initially with X*ba*I and then partially with *Xma*I, yielding the IS*903 *papillation assay plasmid pSKT4.

### Papillation assay

Each papillation assay plasmid was transformed into competent *E. coli *cells prepared and used as described previously [54]. In a standard assay, cells were plated at approximately 100 c.f.u. (colony forming units) per plate. Standard plates contained LB medium [51] supplemented with 1.5% Bacto-agar (Difco/Becton Dickinson and Company, Sparks, MD, USA), 100 μg/ml ampicillin (Ap), 20 μg/ml chloramphenicol (Cm), 0.05% lactose, 40 μg/ml X-gal, and 0.1% arabinose. For the experiments with IS*903 *transposition, glucose replaced arabinose for the adjustment of the transposase expression. In standard assays, the plates were incubated at 30°C for 115 h. To study the suitability of different *E. coli *strains, the effect of arabinose concentration, as well as growth temperature and time, were varied. The effect on papillation of the number of growing colonies per plate was studied by plating cells to produce different colony densities. For each data point, three to six representative colonies were photographed using an Olympus (Tokyo, Japan) ColorView II digital camera attached to an Olympus SZX12 stereomicroscope equipped with Zeiss (Oberkochen, Germany) KL1500 LCD cold light source. The size of the colonies was measured, and the number of papillae in each colony was enumerated manually by the use of AnalySIS software (Soft Imaging System, Olympus). The same imaging system was used for the time-lapsed video recording (Additional file 3).

### Western blotting

The expression of wild-type MuA protein at different arabinose concentrations was determined by analysing whole-cell lysates using sodium dodecyl sulphate-polyacrylamide gel electrophoresis (SDS-PAGE). For protein expression, *E. coli *DH5α(pLHH4) cells were grown in LB-Ap-Cm medium (5 ml) at 37°C to an OD_600 _of 0.5, and MuA expression was induced by the addition of arabinose. Following incubation at 28°C for 2 h, cells from 0.5 ml of the culture were pelleted and resuspended in 150 μl of 1× SB loading buffer (100 mM Tris·HCl, pH 6.8, 200 mM dithiothreitol, 4% SDS, 0.2% bromophenol blue, 20% wt/wt glycerol). The proteins (5 μl of 1:8 diluted sample in 1× SB) were separated on a 10% SDS-PAGE gel and blotted onto Immobilon™P polyvinylidene difluoride membrane (Millipore, Billerica, MD, USA) using the Mini Trans-Blot Electrophoretic Transfer Cell (Bio-Rad, Hercules, CA, USA), according to the manufacturer's instructions. The membrane was blocked at room temperature overnight using 5% skim milk powder dissolved in phosphate-buffered saline (PBS) buffer [51]. Following a wash with PBS, the membrane was probed with the primary rabbit anti-MuA antibody (1:1,000 dilution) and subsequently with the secondary horseradish peroxidase-conjugated anti-rabbit (from donkey; Amersham Biosciences/GE Healthcare, Buckinghamshire, United Kingdom) Ig antibody (1:10,000 dilution). The bound peroxidase was detected using the ECL Plus Western Blotting System (Amersham Biosciences/GE Healthcare,) as specified by the supplier. Immunoreactive bands were visualized using a Fujifilm LAS-3000 system (Fujifilm Corporation, Tokyo, Japan).

## Competing interests

The authors declare that they have no competing interests.

## Authors' contributions

MIP, TSR, LJH, AL, SH-P and SK carried out the experiments. MIP, AL, SH-P and HS conceived the experiments. MIP, TSR, SK and HS wrote the manuscript.

## Supplementary Material

Additional file 1 **Supp. Table 1. *E. coli *strains**. *E. coli *strains used in the work. Click here for file

Additional file 2 **Supp. Fig. 1. Dependency of papillae number per colony on the number of growing colonies**. Bacterial colonies were grown at different densities (upper left corner) on a standard 9-cm plate. Colony size and the corresponding number of papillae were determined. (a) Strain DH10B. (b) Strain DH5α. Click here for file

Additional file 3 **Supp. Video 1. Time-lapsed video of papillation assay**. Shown are bacterial colonies growing on a standard papillation medium containing 1 × 10^-4 ^% arabinose. The standard X-gal concentration was increased to 120 μg/ml to enhance contrast. The plates were incubated initially at 25°C for 46.5 h in an unilluminated chamber. Subsequently, for recording, the plates were transferred under a microscope with an artificial LCD light source. The recording was done at ~22°C with 1-h time lapse intervals for 6 days (144 h). The colonies shown represent wild-type MuA (Wild type) and MuA_63-663 _(Del-N). Click here for file

Additional file 4 **Supp. Fig. 2. Universal papillation assay plasmid pSKT1**. This plasmid is identical to pLHH4 (see Figure 1a), except that the MuA transposase gene and Mu R-ends are replaced by polylinkers (*Nco*I 319-*Hin*dIII 467, *Spe*I 3560-*Not*I 3567, *Xma*I 7777-*Xba*I 7789, respectively). Unique restriction sites are shown in red font. Click here for file

Additional file 5 **Supp. Fig. 3. Papillation assay plasmid pSKT4 for IS*903 *transposition**. This plasmid contains the IS*903 *transposase gene and the transposon ends within the polylinkers of pSKT1 (see Additional file 4). Click here for file

Additional file 6 **Supp. Table 2. Plasmids**. Plasmids used in the work. Click here for file

Additional file 7 **Supp. Table 3. Oligonucleotides**. Oligonucleotides used in the work. Click here for file
