# Supplementary material for: Universal platform for quantitative analysis of DNA transposition
Source: Mob DNA. 2010 Nov 26;1:24. doi: 10.1186/1759-8753-1-24 (PMC3003695; doi:10.1186/1759-8753-1-24)
Supplement: Additional file 1 — Supp. Table 1. E. coli strains. E. coli strains used in the work. [file 1759-8753-1-24-S1.DOC]

**Additional file 1. *E.coli* strains.**

| Strain | Genotype | Reference |
| --- | --- | --- |
|  |  |  |
| DH10B | F^−^ *endA1 recA1 galE15 galK16 nupG rpsL ΔlacX74 φ80ΔlacZΔM15 araD139 Δ(ara, leu)7697 mcrA Δ(mrr-hsdRM-mrcBC)* | [55] |
| DH5α | F^−^ *endA1 supE44 thi-1 recA1 relA1 gyrA96 deoR nupG φ80ΔlacZΔM15 Δ(lacZYA-argF)U169 hsdR17(r_k_^−^ m_k_^+^)* | Invitrogen |
| JM109 | *recA1 endA1 supE44 thi-1 relA1 gyrA96* Δ(*lac-proAB*) *hsdR17*(*r_k_^−^ m_k_^+^*) [F´ *traΔ36 proAB^+^ lacI*^q^*Z*Δ*M15*] | [56] |
| HT321^a^ | *endA1 supE44 thi-1 relA1 gyrA96* Δ(*lac-proAB*) *hsdR17*(*r_k_^−^ m_k_^+^*) [F´ *traΔ36 proAB^+^ lacI*^q^*Z*Δ*M15*] *pcnB::Tn10*(Tc^R^) | [33] |
|  |  |  |

^a^ JM107 [56] derivative
