# Supplementary figures and images for: Universal platform for quantitative analysis of DNA transposition
Source: Mob DNA. 2010 Nov 26;1:24. doi: 10.1186/1759-8753-1-24 (PMC3003695; doi:10.1186/1759-8753-1-24)

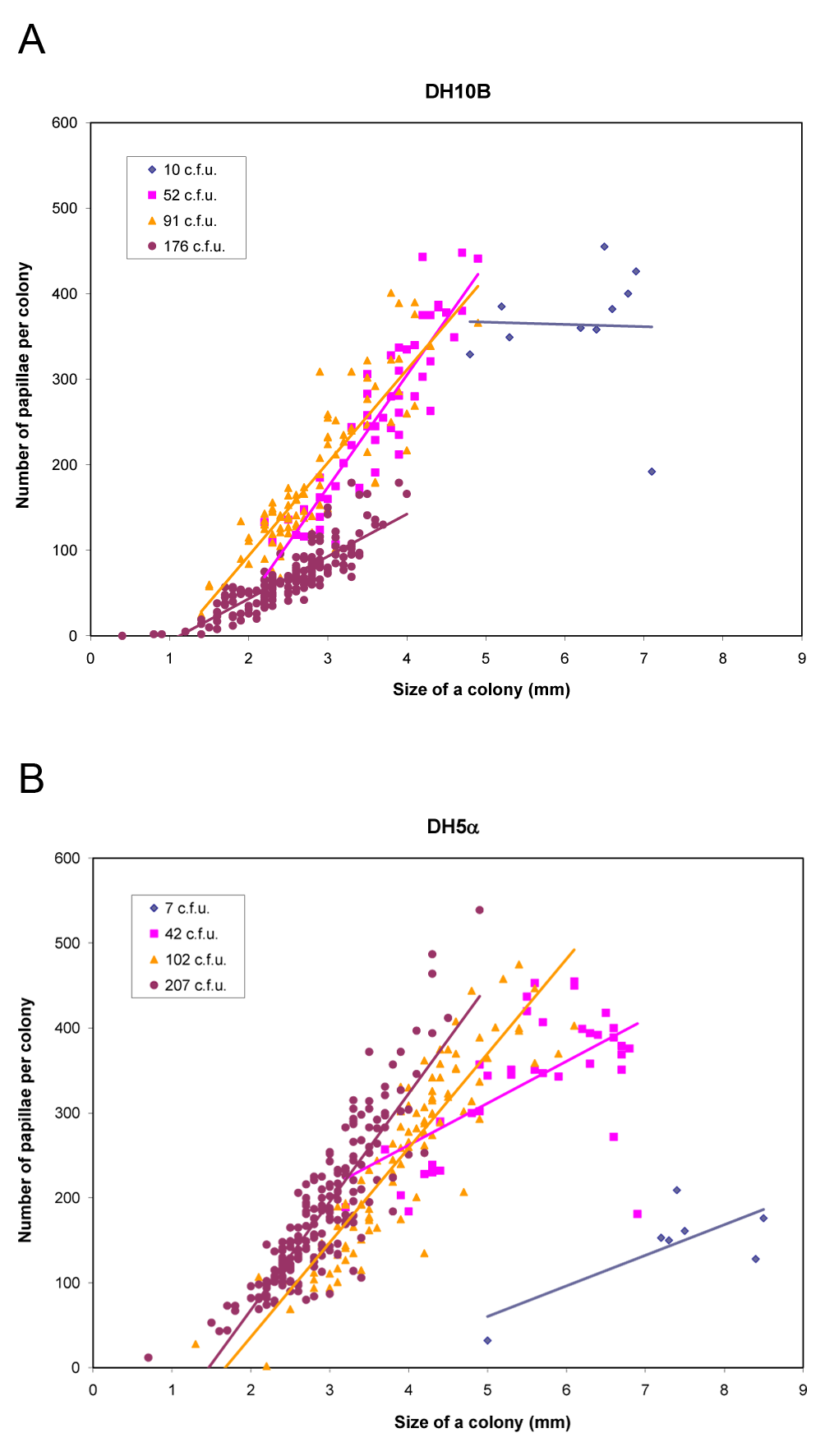

Supplement: Additional file 2 — Supp. Fig. 1. Dependency of papillae number per colony on the number of growing colonies. Bacterial colonies were grown at different densities (upper left corner) on a standard 9-cm plate. Colony size and the corresponding number of papillae were determined. (a) Strain DH10B. (b) Strain DH5α. [file 1759-8753-1-24-S2.TIFF]

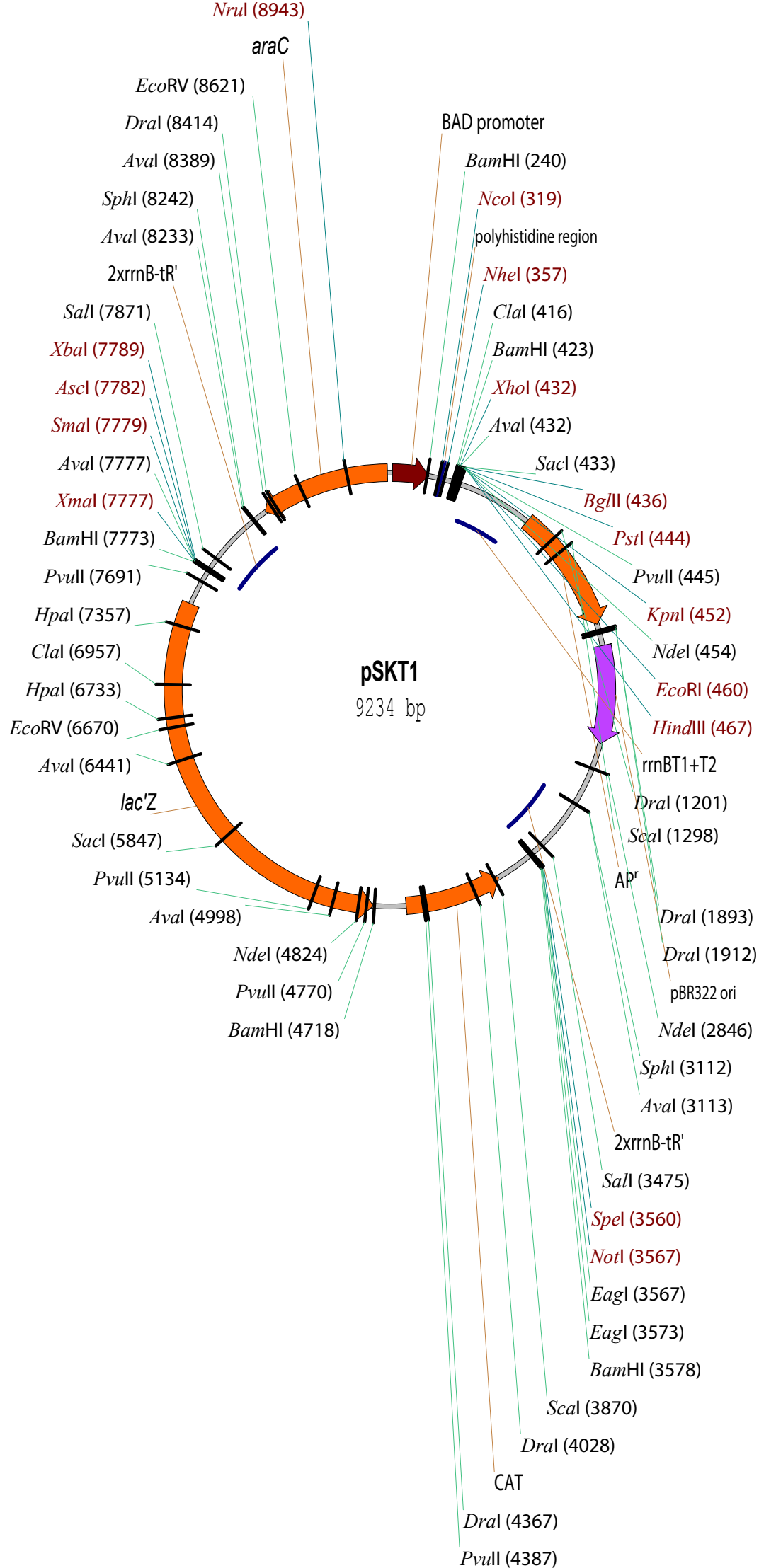

Supplement: Additional file 4 — Supp. Fig. 2. Universal papillation assay plasmid pSKT1. This plasmid is identical to pLHH4 (see Figure 1a), except that the MuA transposase gene and Mu R-ends are replaced by polylinkers (NcoI 319-HindIII 467, SpeI 3560-NotI 3567, XmaI 7777-XbaI 7789, respectively). Unique restriction sites are shown in red font. [file 1759-8753-1-24-S4.PDF]

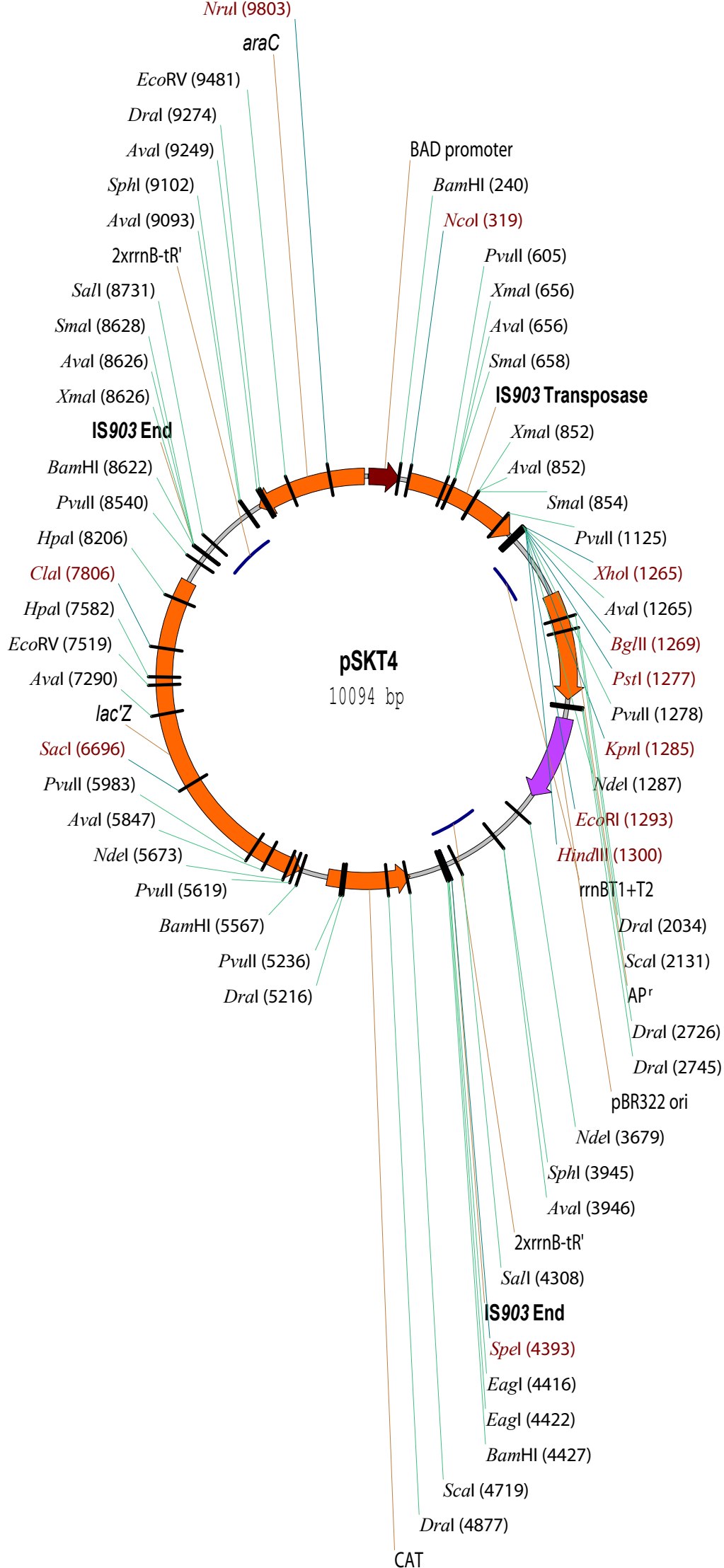

Supplement: Additional file 5 — Supp. Fig. 3. Papillation assay plasmid pSKT4 for IS903 transposition. This plasmid contains the IS903 transposase gene and the transposon ends within the polylinkers of pSKT1 (see Additional file 4). [file 1759-8753-1-24-S5.PDF]
