# Supplementary material for: Universal platform for quantitative analysis of DNA transposition
Source: Mob DNA. 2010 Nov 26;1:24. doi: 10.1186/1759-8753-1-24 (PMC3003695; doi:10.1186/1759-8753-1-24)
Supplement: Additional file 6 — Supp. Table 2. Plasmids. Plasmids used in the work. [file 1759-8753-1-24-S6.DOC]

**Additional file 6. Plasmids.**

| Plasmid | Relevant characteristics | Marker | Reference |
| --- | --- | --- | --- |
|  |  |  |  |
| pNT105 | pACYC184 derivative expressing IS*903* transposase and carrying a *Δ_1-8_lacZ*-containing transposon with IS*903* ends | Cm^R^ | [57] |
| pSupF-Mu | pUC19 derivative, carrier plasmid for *supF*-containing mini-Mu transposon | Ap^R^ | [23] |
| pBC SK(+) | source of *cat* gene | Cm^R^ | Stratagene |
| pBADHisA | source of *rrnB* T1+T2, pBR322 ori | Ap^R^ | Invitrogen |
| pMK591 | pET-3d derivative expressing MuA | Ap^R^ | [58] |
| pMK616 | pET-3d derivative expressing MuA_E392Q_ | Ap^R^ | [37] |
| pALH6 | *MuA* gene from pMK591 cloned between *Nco*I and *Bam*HI sites of pBADHisA | Ap^R^ | [59] |
| pTLH1 | pBADHisA derivative carrying a mini-Mu transposon containing *Δ_1-8_lacZ* and *cat* genes | Ap^R^, Cm^R^ | This work |
| pLHH4 | pTLH1 derivative expressing MuA transposase, Mu R-ends | Ap^R^, Cm^R^ | This work |
| pLHH12 | pTLH1 derivative expressing MuA_E392Q_ transposase, Mu R-ends | Ap^R^, Cm^R^ | This work |
| pMPH14 | pTLH1 derivative expressing MuA_77-663_ transposase, Mu R-ends | Ap^R^, Cm^R^ | This work |
| pMPH17 | pTLH1 derivative expressing MuA_1-615_ transposase, Mu R-ends | Ap^R^, Cm^R^ | This work |
| pMPH18 | pTLH1 derivative expressing MuA_77-615_ transposase, Mu R-ends | Ap^R^, Cm^R^ | This work |
| pMPH23B | pTLH1 derivative expressing MuA transposase, no ends | Ap^R^, Cm^R^ | This work |
| pSKT1 | pTLH1 derivative carrying three polylinkers | Ap^R^, Cm^R^ | This work |
| pSKT4 | pTLH1 derivative expressing IS*903* transposase, IS*903* ends | Ap^R^, Cm^R^ | This work |
