# Supplementary material for: Universal platform for quantitative analysis of DNA transposition
Source: Mob DNA. 2010 Nov 26;1:24. doi: 10.1186/1759-8753-1-24 (PMC3003695; doi:10.1186/1759-8753-1-24)
Supplement: Additional file 7 — Supp. Table 3. Oligonucleotides. Oligonucleotides used in the work. [file 1759-8753-1-24-S7.DOC]

**Additional file 7.** **Oligonucleotides.**

| Application | Name | Sequence | Description |
| --- | --- | --- | --- |
| *Δ_1-8_lacZ* | HSP173 | GCGCGCGGATCCTTATTTTTGACACCAGACCAACTGG | PCR, marker gene from pNT105, include *Bam*HI site for cloning |
|  | HSP408 | GCGCGCGGATCCCGTCGTTTTACAACGTCG |  |
| *cat* | HSP360 | GCGCGCGGATCCTTTTCGACCGAATAAATACC | PCR, antibiotic cassette from pBC SK(+), include *Bam*HI site for cloning |
|  | HSP361 | GCGCGCGGATCCTATCGTCAATTATTACCTCC |  |
| *rrnB* T1+T2 | HSP356 | GCCAATAAGCATGCTCGGGATAAAACAGAATTTGCCTG | PCR, terminators from pBADHisA, include *Acc*I or *Sph*I site for cloning |
|  | HSP357 | GGGCATTTGTCGACGAGTTTGTAGAAACGCAAAAAGG |  |
| λtR’ | HSP352 | CGACAAATGCAATCCCGAAACAGTTCGCAGGTAATAGTTAGAGCCTGCATAACGGTTTCGGGATTTTTTATATCTGCG | Annealed to generate a terminator, *Acc*I and *Bam*HI compatible sticky ends |
|  | HSP353 | GATCCGCAGATATAAAAAATCCCGAAACCGTTATGCAGGCTCTAACTATTACCTGCGAACTGTTTCGGGATTGCATTTGT |  |
| *MuA_77-663_* | HSP669 | CGCGCGCCATGGTCGCCCGCCCCACGCTGG | PCR, transposase deletion variant (pALH6 template), include *Nco*I or *Xho*I site for cloning |
|  | HSP351 | GGGCGGCTCGAGTTAAATGGCTTTTTTACGTCTGTTCTGTTCC |  |
| *MuA_1-615_* | HSP184 | CGCGCGCCATGGAACTTTGGGTATCACCGAAAGAG | as above |
|  | HSP681 | GGGCGGCTCGAGTCATGATTCTGGTGCTGCTGG |  |
| *MuA_77-615_* | HSP669 | CGCGCGCCATGGTCGCCCGCCCCACGCTGG | as above |
|  | HSP681 | GGGCGGCTCGAGTCATGATTCTGGTGCTGCTGG |  |
| *Δ_1-8_lacZ cat* | HSP689 | CGCAGATCTCTAGAGGCGCGCCCCCGGGATCCCGTCGTTTTACAAC | PCR, reporter region from pLHH2, include *Bgl*II site for cloning, additional linker sites *Not*I and *Spe*I or *Xba*I and *Xma*I |
|  | HSP685 | GCGCAGATCTACTAGTGCGGCCGCGGCCGGATCCTATCGTCAATTATTA |  |
| Mu R-ends | HSP686 | CTAGTGAAGCGGCGCACGAAAAACGCGAAAGCGTTTCACGATAAATGCGAAAAC | Annealed to generate Mu R-ends with flanking *Not*I and *Spe*I (HSP686/HSP687) or *Xba*I and *Xma*I (HSP686/688) compatible sticky ends |
|  | HSP687 | GGCCGTTTTCGCATTTATCGTGAAACGCTTTCGCGTTTTTCGTGCGCCGCTTCA |  |
|  | HSP688 | CCGGGTTTTCGCATTTATCGTGAAACGCTTTCGCGTTTTTCGTGCGCCGCTTCA |  |
| IS903 ends | HSP690 | CTAGTGGCTTTGTTGAATAAATC | Annealed to generate IS903 ends with flanking *Not*I and *Spe*I or *Xba*I and *Xma*I |
|  | HSP691 | GGCCGATTTATTCAACAAAGCCA |  |
|  | HSP692 | CCGGGATTTATTCAACAAAGCCA |  |
| IS903 transposase | HSP693 | CGCGCGCCATGGCAAAGCAAAAGTTCAAAATCAC | PCR, IS903 transposase, include *Nco*I and *Xho*I sites for cloning |
|  | HSP694 | GGGCGGCTCGAGTTCCGGGGTTGTGTTTTCAG |  |
